# Bilateral neuromuscular control in patients one year after unilateral ACL rupture or reconstruction. A cross-sectional study

**DOI:** 10.1016/j.heliyon.2024.e24364

**Published:** 2024-01-11

**Authors:** Angela Blasimann, Aglaja Busch, Philipp Henle, Sven Bruhn, Dirk Vissers, Heiner Baur

**Affiliations:** a Bern University of Applied Sciences, School of Health Professions, Division of Physiotherapy, 3008, Bern, Switzerland; b University of Antwerp, Faculty of Medicine and Health Sciences, Department of Rehabilitation Sciences and Physiotherapy, 2610, Wilrijk, Belgium; c University of Potsdam, University Outpatient Clinic, Sports Medicine & Sports Orthopedics, 14469, Potsdam, Germany; d Lindenhof Group AG, Sonnenhof Orthopaedic Center, 3006, Bern, Switzerland; e University of Bern, Bern University Hospital, Inselspital, Department of Orthopaedic Surgery and Traumatology, 3010, Bern, Switzerland; f University of Rostock, Institute of Sports Science, 18051, Rostock, Germany

**Keywords:** Anterior cruciate ligament, ACL, Knee injuries, Reconstruction, Conservative treatment, Rehabilitation, Physiotherapy, Neuromuscular control, Electromyography, EMG

## Abstract

**Objectives:**

To compare bilateral neuromuscular control in patients one year after anterior cruciate ligament reconstruction (ACL-R) or conservative treatment (ACL-C) to healthy controls (ACL-I).

**Design:**

Cross-sectional study.

**Setting:**

Electromyography of vastus medialis (VM) and lateralis (VL), biceps femoris (BF) and semitendinosus (ST) was recorded during stair descent and anterior tibial translation. Each step of stair descent was divided into pre-activity, weight-acceptance and push-off phase. Pre-activation, short, medium (MLR) and long latency responses (LLR) were defined for reflex activity.

**Participants:**

N = 38 patients one year after ACL reconstruction (ACL-R), N = 26 participants with conservative treatment one year after ACL rupture (ACL-C), N = 38 healthy controls with an intact ACL (ACL-I).

**Main outcome measures:**

Normalized root mean squares per muscle and phase (α = 0.05).

**Results:**

During stair descent, within-group leg differences were found for the quadriceps in ACL-R during all phases and for the BF in ACL-C during weight-acceptance. Between-group leg differences were found for BF in both patient groups compared to ACL-I during push-off.

Between-group differences in pre-activation for VM between ACL-R and ACL-C, and between ACL-C and ACL-I were found, and as LLR between patients and ACL-R versus ACL-I. Pre-activation of BF and MLR of ST differed for each patient group compared to ACL-I.

**Conclusions:**

Bilateral neuromuscular alterations are still present one year after ACL rupture or reconstruction.

## 1 Introduction

Ruptures of the anterior cruciate ligament (ACL) are multifactorial [1] and are mainly classified as non-contact injuries [2]. Most non-contact ACL ruptures happen shortly (17–50 ms) after initial foot-ground contact within a time frame which is too short for adequate mechanosensory feedback (e.g., reflex response) [2].

ACL ruptures can be treated surgically or conservatively, depending on the patient's activity level, type of sports and occupation, former knee injuries etc. [3]. At 2- and 5-year follow-up, no differences in patient-reported knee function and incidence of knee osteoarthritis between ACL patients with early reconstruction, conservative treatment followed by reconstruction or conservative treatment alone were found [4]. Nonetheless, this study was limited in reported functional outcomes and demands for further research.

Altered sensorimotor control and neuromuscular adaptations in patients after ACL reconstruction have been stated previously [5]. Deficits in voluntary activation with medium to large effect sizes and limited to moderate evidence as well as increased long-term spinal excitability with limited effect sizes and strong evidence have been summarized [6]. Neuromuscular alterations have been reported to be bilateral in various functional tasks [[7], [8], [9]]. However, these studies are limited to either reconstructed or conservatively treated patients with an ACL rupture and may vary greatly in their included measurement time points.

Therefore, the purpose of this cross-sectional study was to investigate the neuromuscular activity in the involved and non-involved leg of participants one year after surgical or conservative treatment of an ACL rupture compared to a healthy control group during stair descent and artificially induced anterior tibial translation. Based on literature, we hypothesized that altered neuromuscular control in the form of lower activity of the quadriceps and of higher activity of the hamstrings is present in the involved as well as in the contralateral leg of patients after an ACL reconstruction (ACL-R) or conservative treatment (ACL-C) compared to healthy controls with an intact ACL (ACL-I) [8,9].

## 2 Methods

### 2.1 Study design and sample size calculation

A cross-sectional, experimental study design with two patient groups and a healthy asymptomatic control group was determined to investigate neuromuscular control one year after an ACL reconstruction or conservative treatment or rehabilitation alone. One group consisted of participants after an ACL reconstruction (ACL-R), one of participants with conservative treatment (ACL-C) after ACL rupture, and one of healthy controls with an intact ACL (ACL-I).

A priori sample size calculation based on own pilot data [7] revealed a sample size for independent comparisons of n = 10 participants per group (α = 0.05, actual power: 0.96, effect size d: 1.78; G*Power, version 3.1, Heinrich-Heine University, Düsseldorf, Germany) [10].

### 2.2 Participants

Participants for the ACL-R and ACL-C group had been recruited via an orthopaedic surgeon, private physiotherapy practices or sports clubs between January 2018 and August 2021. Healthy people for the control group (ACL-I) had been recruited from local sport clubs, and among members of the Bern University of Applied Sciences (Switzerland). Matching was based on sex, age, body height, body mass and dominant leg, defined as the preferred leg to kick a ball with [11]. The following inclusion criteria were applicable for all participants: age between 16 and 60 years, physically active at least 2x/week for 45 min [12], Tegner activity score of ≥5 [13]. Magnetic resonance imaging was available for participants in the ACL-R or ACL-C group. In those groups, time since rupture or reconstruction had to be between 11 and 14 months. Any type of ACL reconstruction was included. Exclusion criteria for all participants were former knee pathology, other injury of the lower extremity, back pain, musculoskeletal disorders refraining from test protocol, cardiac, neurological or peripheral vascular disease, acute infection, alcohol abuse, current pain medication, thrombosis, pregnancy, dementia, and not being able to understand written or oral German.

**Fig. S1:**
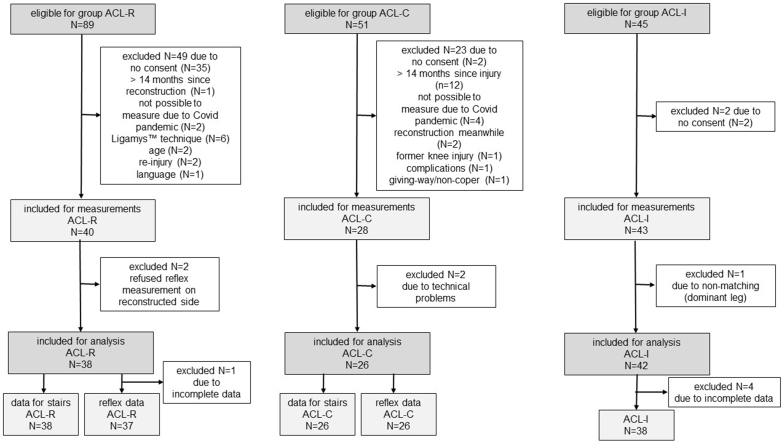
Flowchart of eligibility, recruitment process and inclusion of participants for all three groups Legend (Fig. S1): ACL-C = anterior cruciate ligament rupture conservatively treated; ACL-I = anterior cruciate ligament intact (= healthy controls); ACL-R = anterior cruciate ligament reconstructed (=patients); N = number of.

In total, N = 185 people volunteered for this study and were assigned to one of the three groups, based on ACL status. Data from 38 subjects each for the ACL-R and ACL-I groups as well as 26 subjects for the ACL-C were included in the final analysis. Details regarding the recruitment process can be found in Figure S1.

### 2.3 Measurements

All measurements took place at the Bern Movement Lab (Bern University of Applied Sciences, Bern, Switzerland) by using a formerly described setup [7,14].

In brief, clinical examination, anthropometric data, limb dominance, type of sports, number of hours per week of physical activity, Tegner activity score [13] and Knee Osteoarthritis Outcome Score (KOOS) [15] were collected as described before [14]. The self-adhesive EMG electrodes (Ambu BlueSensor, Ambu A/S, Ballerup, DK; Type P-00-S, inter-electrode distance: 20 mm) were applied on the vastus medialis (VM), vastus lateralis (VL), semitendinosus (ST) and biceps femoris (BF) muscle on both limbs according to SENIAM preparation standards and guidelines [16] including a reference electrode placed on the right patella. Inter-electrode impedance was controlled and kept ≤2 kΩ (D175 Electrode Impedance Meter, Digitimer, Herfordshire, UK). Before and after the measurements, the actual wellbeing and pain were assessed by using a visual analogue scale (VAS) [17].

The measurements started with a warm-up on an instrumented treadmill (quasar med, h/p/cosmos sports & medical GmbH, Nussdorf-Traunstein, D) for 10 min at 1.39 m/s (5 km/h) and additional 2 min treadmill-walking at the same speed with recordings of the EMG signals for submaximal EMG normalization [14]. The initial contact of each gait cycle was detected by two force transducers (type KMB52, 10 kN, MEGATRON Elektronik GmbH & Co. KG, Putzbrunn, D) mounted under the treadmill. Afterwards, each participant completed two experimental situations in the same order: stair descent and stretch reflex measurements [14].

### 2.4 Stair descent

Participants descended a custom-made wooden stairway with six steps ten times at self-selected speed without using the railings [14]. To identify gait cycles, the stairway had two embedded multicomponent force plates in the third and fourth step. Stair descent was divided into three movement cycles: the pre-activation phase (PRE) covered 150 ms prior to initial foot-floor contact until initial contact. The weight acceptance phase (WA) included initial contact until the lowest applied vertical ground reaction force was reached. The push-off phase (PO), followed the WA phase until vertical ground reaction forces declined to zero [14].

### 2.5 Stretch reflex measurements

To assess stretch reflexes, artificial anterior tibial translation was applied on both legs in randomized order. Starting position was standing upright with the hands placed at the pelvis, knees in 30° flexion and equal body mass distribution, controlled by bipedal stand on two force plates [14]. To be blinded to the time point of artificially induced anterior tibial translation and to avoid any acoustic anticipation, participants wore headphones with music and ear protection.

A rope and pulley system applied a standardized impulse to the tibia shank inducing an anterior tibial translation [18]. Onset of the applied force monitored by a force transducer was considered as the trigger signal for the anterior tibial translation [18]. Further details describing the setup can be found elsewhere [14,18]. Artificial tibial translation was elicited in two series with 15 repetitions per lower extremity. Between the series, the participants were given a short break to minimize fatigue or excessive co-contraction. Four pre-defined time intervals were used as outcomes: -50-0 ms pre-activation (PRE_50), 20–40 ms short latency response (SLR), 40–60 ms medium latency response (MLR) and 60–95 ms long latency response (LLR) [14,19].

### 2.6 Signal transmission, data processing and normalization

Since all force sensor signals from treadmill, stairway and rope-pulley system were recorded together with the EMG signals in one LabVIEW-based software (IMAGO Record, pfitec, Endingen, D), no delay due to synchronization was present. Therefore, no later synchronization during the post-processing was needed.

First, EMG signals were transmitted across a differential-preamplifier to a telemetric main amplifier (PowerPack, pfitec, Endingen, D), band-pass filtered at 10 Hz–1000 Hz and recorded at 2000 Hz for treadmill walking and stair descent, and at 4000 Hz for the stretch-reflex measurement [20]. Afterwards, the signals were converted from analogue to digital (type NI PCI 6255, National Instruments, Austin, USA), registered and further processed with the same LabVIEW-based software (IMAGO Record, pfitec, Endingen, D). Then, all raw EMG signals were full wave rectified. Additionally, raw EMG signals from treadmill walking and stair descent were band-pass filtered at 10–500Hz (Butterworth, 2nd order). For the defined time windows as described above, RMS values were calculated and exported in Excel spreadsheets (Windows 10, Microsoft Corporation, Redmond WA, USA). Individual means out of 30 gait cycles (treadmill walking), ten steps (stair descent), and 30 tibia translations per extremity for each muscle in each time interval were calculated. RMS values were normalized according to the corresponding time intervals retrieved during treadmill walking (100 % of neuromuscular activity) and used for comparability within subjects. Reflex responses during artificial tibial translation, and activations during stair descent were normalized and expressed as a percentage (% EMG) of respective treadmill walking activity [21,22].

### 2.7 Statistical analysis

Data analysis was performed by using the Statistical Package for the Social Science (SPSS) software (SPSS Statistics for Windows, version 28.0, IBM, Armonk NY, USA). The level of significance was set at 0.05. Participants’ characteristics (anthropometric data, physical activity, KOOS, TAS) were tested for normal distribution by using Kolmogorov-Smirnov test, followed by non-parametric t-tests (Mann-Whitney-U and Kruskal-Wallis) to detect differences between the groups (ACL-R, ACL-C and ACL-I). To evaluate group differences of the neuromuscular activity, all EMG outcomes of each muscle (VM, VL, BF, ST) per phase during stair descent (PRE, WA, PO) and artificial tibial translation (PRE_50, SLR, MLR, LLR) were analyzed separately in the involved (reconstructed, conservatively treated, or matched knee based on side of injury) and the non-involved leg (contralateral knee). The involved leg of an ACL-I participant was determined according to the injured side of the matching patient. Kruskal-Wallis tests were used to evaluate group differences since the requirements for a parametric procedure were not fulfilled (Shapiro Wilk and Levene test). For pair-wise post-hoc comparisons, the Mann-Whitney-U test for independent samples and for intra-individual leg comparison, the Wilcoxon test for dependent samples was carried out, including Dunn Bonferroni correction for multiple testing. Although not all data was normally distributed, mean values and standard deviations (SD) are reported to allow comparison with other studies.

Effect sizes (ES) were calculated based on Pearson's correlation [23]. An ES below 0.3 was interpreted as small effect, 0.3 ≤ *r* < 0.5 as medium effect, and equal to 0.5 or higher as large effect [24].

## 3 Results

### 3.1 Characteristics of participants

Patient groups had significantly higher weekly hours of physical activity (p = 0.014, 0.011 respectively) and activity levels (p = 0.001, p < 0.0001 respectively) compared to the healthy controls (ACL-I). No other significant differences were found for anthropometric data (Table 1). Details of concomitant injuries, type of treatment and autograft for reconstruction (if applicable) are described in Table S1.

**Table 1:** Characteristics of 38 participants with an ACL reconstruction (ACL-R), 26 participants with a conservatively treated ACL rupture (ACL-C) and 38 healthy controls with an intact ACL (ACL-I), matched by sex, age, body height, body mass and leg dominance.

	Mean ± SD if not otherwise stated			p-value			
Characteristics	ACL-R N = 38	ACL-C N = 26	ACL-I N = 38	ACL-R vs. ACL-I	ACL-C vs. ACL-I	ACL-R vs. ACL-C	overall
**Age [years]**	32.02 ± 12.21	38.38 ± 11.65	33.13 ± 9.16	0.391	0.099	0.031*	0.074
**Body height [cm]**	173.55 ± 6.25	170.23 ± 7.59	173.66 ± 6.96	0.831	0.033*	0.075	0.087
**Body mass [kg]**	71.71 ± 11.19	71.06 ± 14.88	68.38 ± 9.43	0.244	0.942	0.456	0.507
**BMI [kg/m**^**2**^**]**	23.94 ± 2.73	24.47 ± 4.64	22.64 ± 2.02	0.015*	0.305	0.305	0.056
**Time since injury (months)**	12.7 ± 1.4	12.5 ± 1.1	–	<0.0001*	–	0.579	–
**Sex: Ratio of ♀:♂ (%)**	17:21 (44.7:55.3)	16:10 (61.5:38.5)	20:18 (52.6:47.4)	0.494	0.848	0.190	0.419
**Leg dominance (right:left)**	35:3	23:3	34:4	0.694	0.899	0.626	0.876
**Prehabilitation (yes:no)**	13:25	–	–	<0.0001*	–	<0.0001*	–
**PT after surgery/injury (yes:no)**	38:0	26:0	–	<0.0001*	–	<0.0001*	–
**Physical activity [min/week]**	425.96 ± 265.10	373.02 ± 158.18	293.26 ± 182.81	0.014*	0.011	0.811	0.014*
**Tegner score (max. 10 points)**	6.71 ± 1.45	6.96 ± 1.18	5.53 ± 1.31	0.001*	<0.0001*	0.273	<0.0001*
**KOOS subscale (absolute values)**							
pain (9 items, max. 36 p.)	4.47 ± 3.32	3.35 ± 2.99	0.37 ± 0.71	<0.0001*	<0.0001*	0.119	<0.0001*
other symptoms (7 items, max. 28 p.)	5.95 ± 3.80	5.08 ± 3.12	1.58 ± 1.52	<0.0001*	<0.0001*	0.532	<0.0001*
ADL (17 items, max. 68 p.)	2.50 ± 3.42	2.23 ± 4.03	0.08 ± 0.36	<0.0001*	<0.0001*	0.380	<0.0001*
sports & leisure (5 items, max. 20 p.)	3.63 ± 2.88	2.69 ± 2.77	0.21 ± 0.70	<0.0001*	<0.0001*	0.151	<0.0001*
HRQoL (4 items, max. 16 p.)	4.73 ± 3.29	3.81 ± 2.90	0.37 ± 1.00	<0.0001*	<0.0001*	0.259	<0.0001*
**VAS**							
*wellbeing pre [mm]*	5.53 ± 8.60	5.35 ± 10.31	5.29 ± 6.90	0.643	0.666	0.972	0.870
*wellbeing post [mm]*	7.29 ± 9.00	5.58 ± 9.91	6.55 ± 7.39	0.937	0.355	0.402	0.612
*pain pre [mm]*	3.08 ± 4.08	4.35 ± 8.89	1.08 ± 2.06	0.026*	0.037*	0.977	0.045*
*pain post [mm]*	6.18 ± 13.25	6.42 ± 10.57	3.42 ± 7.88	0.264	0.082	0.486	0.209

Data is presented as mean ± standard deviation (SD) unless otherwise stated. * Indicates significant p-values (p < 0.05); dashed lines indicate not applicable.

Abbreviations: ACL-C = anterior cruciate ligament rupture conservatively treated; ACL-I = anterior cruciate ligament intact (healthy controls); ACL-R = anterior cruciate ligament reconstructed (patients); ADL = activity of daily life; HRQoL = health-related quality of life; KOOS = Knee injury and Osteoarthritis Outcome Score (scoring per item: 0 = no problems; 4 = extreme problems); max. = maximum; p = points; post = after the measurements; pre = before the measurements started; PT = physiotherapy; *Tegner activity score (preinjury) ranging from 0 (sick leave or disability pension) to 10 (competitive sport on a professional level); VAS = visual analogue scale from 0 to 100 mm.

### 3.2 Stair descent

**Fig. 1:**
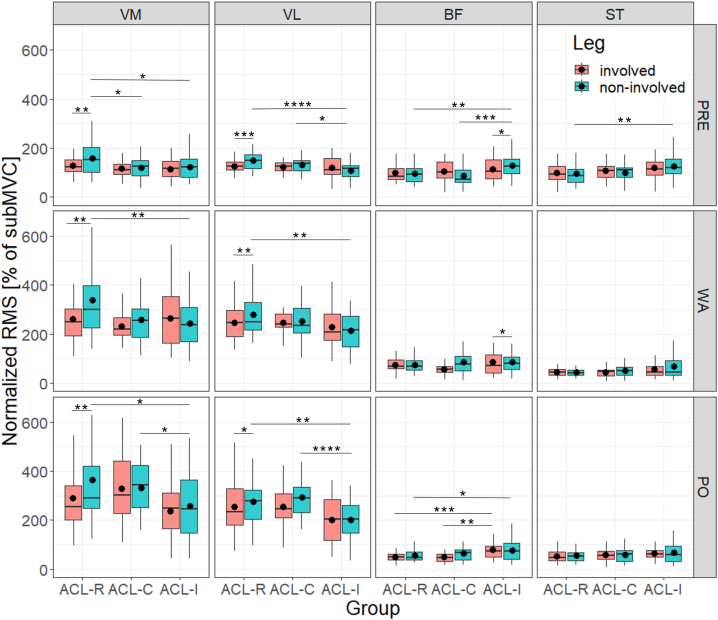
Stair descent: box plots of all four muscles of the involved and non-involved leg for pre-activity, weight-acceptance and push-off phase (from top to bottom). Legend (Fig. 1): results are presented for all three phases of stair descent as median and interquartile ranges including mean value expressed as black dot; *p < 0.05; **p < 0.01; ***p < 0.001; ****p < 0.0001; ACL = anterior cruciate ligament; ACL-C = group with conservative treatment after ACL rupture; ACL-I = healthy controls with intact ACL; ACL-R = group with ACL reconstruction; BF = biceps femoris; involved = formerly injured or reconstructed side, or matched leg of controls (based on side of injury); non-involved = non-injured leg of patients, contralateral knee respectively; PRE = pre-activity; PO = push-off phase during stair descent; RMS = root mean square values; ST = semitendinosus; subMVC = submaximal voluntary contraction (normalized values with treadmill walking = 100 % subMVC); VM = vastus medialis; VL = vastus lateralis; WA = weight-acceptance phase.

Fig. 1 provides box plots for all four muscles and all three phases during stair descent, presented separately for legs and groups. Significant results for involved and non-involved leg comparison between and within groups are summarized below and all mean and SD values as well as results for inferential statistics are presented in Table S2.

#### 3.2.1 Leg comparisons within groups

Intragroup comparisons revealed that neuromuscular activity of the involved compared to non-involved leg of ACL-R was significantly lower for the quadriceps in all three movement phases: during PRE (for VM: Δ-18.7 %, ES 0.34, p = 0.004; for VL: Δ-15.8 %, ES 0.37, p = 0.001), WA (for VM: Δ-22.7 %, ES 0.34, p = 0.004; for VL: Δ-11.2 %, ES 0.35, p = 0.003) and PO (for VM: Δ-20.8 %, ES 0.31, p = 0.008; for VL: Δ-7.9 %, ES 0.28, p = 0.018).

The involved leg of ACL-C group demonstrated significantly lower activities in BF during WA only compared to the contralateral leg (Δ-32.2 %, ES 0.34, p = 0.015).

Significant leg differences of the healthy controls were present in the BF during PRE only (Δ-13.1 %, ES 0.29, p = 0.014).

#### 3.2.2 Leg comparisons between groups

For intergroup comparisons, the following results were found: post-hoc analysis comparing neuromuscular activity revealed significant differences for ACL-R compared to ACL-I in the non-involved leg for both quadriceps and hamstrings for PRE phase: for VM (Δ+29.1 %, ES 0.30, p = 0.011), VL (Δ+38.2 %, ES 0.47, p < 0.0001), BF (Δ-26.7 %, ES 0.36, p = 0.002) and ST (Δ-23.7 %, ES 0.32, p = 0.007). During the WA phase, significantly higher activities in the quadriceps were present in the non-involved leg comparing ACL-R to ACL-I: for VM (Δ+40.1 %, ES 0.31, p = 0.008), and for VL (Δ+30.4 %, ES 0.31, p = 0.007). During the PO phase, significantly higher neuromuscular activity in ACL-R was found in the non-involved leg for the VM (Δ+41.6 %, ES 0.27, p = 0.019) and VL (Δ+37.1 %, ES 0.36, p = 0.002), and decreased neuromuscular activity in ACL-R for BF in both legs (involved: Δ-38.4 %, ES 0.39, p = 0.001; non-involved: Δ-29.5 %, ES 0.29, p = 0.014) compared to ACL-I.

Post-hoc analysis in the non-involved leg revealed significantly higher activities for VL (Δ+21.1 %, ES 0.31, p = 0.016) in ACL-C and significantly lower for BF (Δ-32.5 %, ES 0.42, p = 0.001) for ACL-C compared to ACL-I during PRE. During PO, significantly higher activities were present for VM (Δ+28.5 %, ES 0.26, p = 0.040) and VL (Δ+45.4 %, ES 0.48, p < 0.0001) in the ACL-C group. In the involved leg, significantly lower neuromuscular activity in ACL-C compared to ACL-I was found for BF (Δ-37 %, ES 0.37, p = 0.004).

Post-hoc analysis comparing both former patient groups (ACL-R versus ACL-C) revealed significantly higher neuromuscular activity in VM of the non-involved limb (Δ+31.1 %, ES 0.27, p = 0.030) during PRE for ACL-R compared to ACL-C.

### 3.3 Artificial tibial translation

**Fig. 2:**
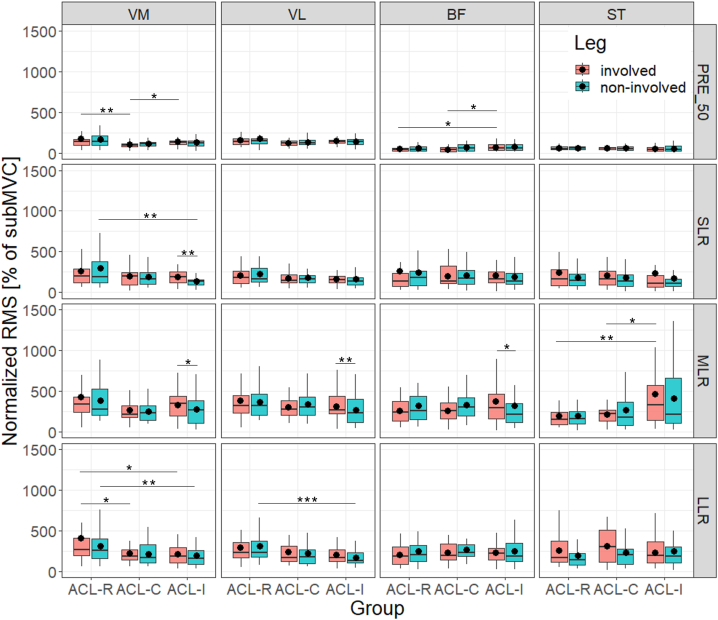
Reflex measurements: box plots of all four muscles of the involved and non-involved leg for pre-activation, short, medium and long latency response during artificial tibia translation (from top to bottom) Legend (Fig. 2): results are presented for all four phases of reflex measurements as median and interquartile ranges including mean value expressed as black dot; *p < 0.05; **p < 0.01; ***p < 0.001; ****p < 0.0001; ACL = anterior cruciate ligament; ACL-C = group with conservative treatment after ACL rupture; ACL-I = healthy controls with intact ACL; ACL-R = group with ACL reconstruction; BF = biceps femoris; involved = formerly injured or reconstructed side, or matched leg of controls (based on side of injury); LLR = long latency response; MLR = medium latency response; non-involved = non-injured leg of patients, contralateral knee respectively; PRE_50 = pre-activation (background activity); RMS = root mean square values; SLR = short latency response; ST = semitendinosus; subMVC = submaximal voluntary contraction (normalized values with treadmill walking = 100 % subMVC); VM = vastus medialis; VL = vastus lateralis.

Fig. 2 provides box plots for all four muscles and all four phases during artificial tibial translation, presented separately for legs and groups. Significant results for the involved and non-involved leg comparison between and within groups are summarized below. All mean and SD values as well as results for inferential statistics are presented in Table S3.

#### 3.3.1 Leg comparisons within groups

In both former patient groups, no significant within-group leg differences were found for any of the investigated muscles and time windows during artificial tibial translation. However, in the group of healthy controls (ACL-I), neuromuscular activity was significantly higher in the matched “involved” leg, based on side of injury, compared to the contralateral leg in two phases: during SLR for VM (Δ+39 %, ES 0.34, p = 0.004) and during MLR for VM (Δ+19.1 %, ES 0.25, p = 0.033), VL (Δ+15.7 %, ES 0.31, p = 0.008) and BF (Δ+18.7 %, ES 0.23, p = 0.044).

#### 3.3.2 Leg comparisons between groups

Post-hoc analysis for intergroup comparisons revealed significant lower pre-activation in the involved leg of ACL-R compared to ACL-I for BF (Δ-28.1 %, ES 0.25, p = 0.035). During SLR, significantly higher activity was found for VM of the non-involved limb of ACL-R (Δ+119.2 %, ES 0.34, p = 0.04). During MLR, ST of the involved leg showed significantly less activity in ACL-R subjects (Δ-57.2 %, ES 0.33, p = 0.007). During LLR, VM of both the involved (Δ+90.9 %, ES 0.28, p = 0.021) and the non-involved leg (Δ+58.5 %, ES 0.31, p = 0.008), and VL of the non-involved leg (Δ+83.1 %, ES 0.38, p = 0.001) presented significantly higher neuromuscular activation in ACL-R compared to ACL-I.

Significantly lower pre-activation in the involved leg for VM (Δ-27.5 %, ES 0.31, p = 0.013) and BF (Δ-36.2 %, ES 0.29, p = 0.022) as well as during MLR for ST (Δ-53.7 %, ES 0.27, p = 0.037) was present in the ACL-C compared to ACL-I.

Post-hoc analysis comparing both former patient groups revealed significantly higher neuromuscular pre-activation for VM of the involved limb (Δ+73.5 %, ES 0.35, p = 0.007) and higher neuromuscular activation during LLR (Δ+84.4 %, ES 0.28, p = 0.034) in patients after ACL reconstruction.

## 4 Discussion

This cross-sectional study compared bilateral neuromuscular activity in patients one year after an ACL rupture with surgical reconstruction or conservative treatment in comparison to a healthy control group during stair descent and an artificially induced anterior tibial translation. One year after an ACL rupture, neuromuscular alterations were still present in both legs of ACL patients, regardless of treatment option.

Reported results are in line with former research [7,14] representing a large range of EMG activity patterns within and between groups in the investigated timeframes and movement phases.

The hypothesis that neuromuscular control would be altered – lower quadriceps and higher hamstrings activity during voluntary activation – in the involved and non-involved leg even one year after an ACL reconstruction in comparison to healthy controls with an intact ACL had been partially confirmed. This altered muscle activity strategy has been described as arthrogenic muscle response meaning a natural mechanism of reflex inhibition and/or muscular facilitation to stabilize and therefore protect the injured joint [25]. In patients with an ACL injury, arthrogenic muscle response might be due to a loss of mechanoreceptors by the ruptured ACL and altered discharge of sensory receptors induced by inflammatory signs and joint laxity [25]. This arthrogenic muscle response with decrease of the quadriceps activity (as ACL antagonist) and increased excitability of the hamstrings (as ACL agonists) reduces potentially dangerous movements for the injured knee joint as it has been shown in ACL-R patients [26]. In the acute phase after ACL rupture or reconstruction, the combination of both low quadriceps and high hamstrings activation in the involved leg compared to the matched leg of the healthy control group could be advantageous as this strategy improves dynamic stability of the knee joint [27]. However, if this altered muscle strategy persists, it might negatively influence joint biomechanics and articular cartilage loading, potentially leading to post-traumatic knee osteoarthritis [8,27].

During stair descent, both legs showed significant less neuromuscular activity of BF during PO. In opposition to our results, athletes after ACL-R (mean time post-surgery 8.5 months) demonstrated lower activity levels of the VM and larger hamstrings activation during a step-down task [9]. However, these participants had ST autografts while participants from our study mainly had quadriceps autografts. Additionally, EMG signals were analyzed in one time interval after step down landings (50–250 ms after initial contact). In the present study, significant differences were found mainly during pre-activity and in the PO phase, where adequate neuromuscular control by the quadriceps is essential to eccentrically decelerate the body weight in knee flexion. It is known that ACL injury may negatively influence intracortical facilitation [28] leading to larger intracortical inhibition which is correlated with decreased capability to voluntarily activate the quadriceps [29]. However, it remains unclear if this strategy with upregulation of the quadriceps and lower activation of the hamstrings has more beneficial long-term effects, because the stress on the ACL in posterior-anterior direction could be increased due to the impaired protective effect by lower hamstrings activation.

Significant interlimb differences during stair descent in ACL-R participants were found, demonstrating decreased activity of the quadriceps in the involved limb compared to the non-involved limb in all examined phases. This may be explained by the artificial injury due to graft harvesting, especially autografts with quadriceps tendon, which must also be compensated accordingly [30]. In our study, two thirds of all patients of the ACL-R group received a quadriceps tendon autograft. Differences between graft types and reconstructive techniques should be investigated to give specific recommendations for individualized rehabilitation [30]. However, further comparisons of graft types were beyond the scope of the present study.

Alterations in neuromuscular activity have been found during artificial tibial translation, as well. From a physiological point of view, the MLR is most relevant as it has been recognized as the most vulnerable phase due to the homonymous interconnection of cruciate ligament receptors and hamstring muscles [18]. This means that the hamstrings need to be specifically targeted during rehabilitation to be sufficiently activated before and during dynamic activities.

Participants after conservative treatment (ACL-C) showed altered neuromuscular control in both legs in both tasks, but not for all muscles and phases. Overall, neuromuscular control in those participants differed less from healthy controls than those of ACL-R participants. Present findings were in line with changes in neuromuscular control of males with an ACL deficiency (mean time since injury 19.8 months) compared to healthy controls during landings from a 30 cm height [31]. The authors reported a reduction in lateral hamstrings activation of the affected leg compared to the matched limb of healthy individuals at SLR [31]. Post-landing EMG of VL was reduced in the involved and non-involved side of ACL deficient participants compared to the control limb during SLR and MLR. Our study confirms findings in the literature demonstrating that current rehabilitation programs may not sufficiently target the impaired neuromuscular control after ACL rupture [32], independently from treatment chosen. During artificial tibial translation, no significant within-group differences were found in both former patient groups, which indicates similar bilateral consequences after ACL rupture – either deterioration or improvement of neuromuscular control. These findings indicate that the non-involved, “healthy” leg is not a good reference to decide upon a safe RTS and does not mean reaching the level of sports ability before ACL rupture [33]. As the function of the contralateral limb worsens in ACL-R patients one and five years after reconstruction [34], lower limb indices could overestimate current sensorimotor competence and therefore should not be used in isolation to evaluate functional performance [33,34]. Ideally, and for athletes probably the case, pre-injury reference values would exist which would consider age, physical and professional activity as well as type and level of sports participation. Moreover, artificial tibial translation gives insight into sensorimotor control mechanisms for knee joint stability [18,19]. This method could help defining outcomes for neuromuscular control to be integrated into current RTS criteria, as has been stated before [35].

### 4.1 Strengths and limitations

There are some strengths and limitations to be considered. This study is one of only a few publications reporting neuromuscular activity of thigh muscles comparing two ACL patient groups after different treatments one year after injury with a healthy control group. According to current knowledge, it is one of the first studies that investigated reflex response after artificially induced tibial perturbation in participants with different treatment modalities after ACL injury.

However, study limitations include the following aspects: as the inclusion of ACL-R participants was not limited to one orthopaedic surgeon and/or one surgical technique, the group presented itself as heterogeneous regarding choice of graft and surgical techniques. Participants were not randomly allocated to either surgical technique or conservative treatment. Therefore, a selection bias cannot be ignored. Furthermore, different treatment modalities (type, duration, content), because no consensus of rehabilitation after ACL rupture exists, might have influenced the results. Moreover, suffering from concomitant injuries was not an exclusion criterion and might have altered or even prolonged rehabilitation. Nonetheless, a narrow time frame for measurements was chosen to reduce these possible influences.

At the time of measurement, all included patients had received medical clearance for RTS. However, not every patient had full, bilaterally comparable active and passive end range of motion in the knee joint. Some ACL-R participants presented with atrophy in the involved, operated leg, reported experiencing painful episodes or were hypermobile in one or both knee joints.

In summary, the heterogeneity within and between patient groups regarding age, physical and mental state, as well as choice of graft, type and duration of rehabilitation, could have influenced the results and limits the generalizability. Finally, the presented methods to assess neuromuscular control are complex and not yet ready to be easily included in physical performance test batteries or used in other clinical settings for follow-up assessment.

## 5 Conclusions

The present study showed that neuromuscular alterations are still present in both lower limbs one year after ACL rupture in comparison to healthy controls, independently from treatment chosen. As both legs are affected, widely used limb symmetry indices should not be used in isolation to decide upon a safe RTS.

Future research should assess both lower limbs and include other outcomes than limb symmetry indices alone when deciding upon a safe return to sports regardless of the treatment path chosen. In addition, it is necessary to evaluate evidence-based, standardized rehabilitation programs for reconstructed and conservatively treated patients. These programs should include neuromuscular, biomechanical, sensorimotor and neurocognitive factors to restore movement quality and performance as had already been stated for ACL reconstruction [32,35]. Moreover, studies with long-term follow-up after clearance for RTS are needed to prove positive effects such as rehabilitation programs with special focus on improvement of neuromuscular control.

## Ethics statement

This study was conducted in accordance with the Declaration of Helsinki and approved by the Ethics Committee of the Canton of Bern (Switzerland), KEK No. 2017–02282. Every participant provided written informed consent.

## Funding

This research was supported by the 10.13039/100000001Swiss National Science Foundation (Bern, Switzerland; project number: 32003B_176060), the Lindenhof Foundation (Bern, Switzerland; project number: 14-10-F) and the Bern University of Applied Sciences, School of Health Professions (Bern, Switzerland; grant for non-tenured staff). All funders had no role in the design of the study, data collection, analysis and interpretation of data, writing the report, and in the decision to submit the manuscript for publication.

## Data availability statement

The data will be provided by the corresponding author upon reasonable request.

## CRediT authorship contribution statement

**Angela Blasimann:** Conceptualization, Data curation, Formal analysis, Investigation, Methodology, Writing – original draft, Writing – review & editing. **Aglaja Busch:** Formal analysis, Investigation, Visualization, Writing – original draft, Writing – review & editing, Data curation, Software, Validation. **Philipp Henle:** Funding acquisition, Resources, Validation, Writing – review & editing, Conceptualization. **Sven Bruhn:** Methodology, Resources, Validation, Writing – review & editing. **Dirk Vissers:** Supervision, Validation, Writing – review & editing, Conceptualization. **Heiner Baur:** Conceptualization, Data curation, Funding acquisition, Methodology, Project administration, Resources, Supervision, Validation, Writing – original draft, Writing – review & editing.

## Declaration of competing interest

The authors declare that they have no known competing financial interests or personal relationships that could have appeared to influence the work reported in this paper.
