# Supplementary material for: Bilateral neuromuscular control in patients one year after unilateral ACL rupture or reconstruction. A cross-sectional study
Source: Heliyon. 2024 Jan 11;10(2):e24364. doi: 10.1016/j.heliyon.2024.e24364 (PMC10803901; doi:10.1016/j.heliyon.2024.e24364)
Supplement: Multimedia component 1 [file mmc1.docx]

Appendix Table A.1: Frequencies of concomitant injuries, types of treatment and graft (if applicable) of participants with an ACL reconstruction (ACL-R) and participants with a conservatively treated ACL rupture (ACL-C).

|  | **ACL-R (N = 38)** | **ACL-C (N = 26)** |
| --- | --- | --- |
| **Medial meniscal tears**  *conservative treatment*  *suture*  *resection*  *none* | 2 (5.3%)  15 (39.5%)  7 (18.4%)  14 (36.8%) | 4 (15.4%)  0  0  22 (84.6%) |
| **Lateral meniscal tears**  *conservative treatment*  *suture*  *resection*  *none* | 1 (2.6%)  8 (21.1%)  1 (2.6%)  28 (73.7%) | 5 (19.2%)  0  0  21 (80.8%) |
| **Medial collateral ligament injury**  *conservative treatment*  *surgery*  *none* | 8 (21.1%)  1 (2.6%)  29 (76.3%) | 8 (30.8%)  0  18 (69.2%) |
| **Lateral collateral ligament injury**  *conservative treatment*  *surgery*  *none* | 2 (5.3%)  0  36 (94.7%) | 4 (15.4%)  0  22 (84.6%) |
| **Bone bruise**  yes  none | 0  38 (100%) | 7 (26.9%)  19 (73.1%) |
| **Cartilage defect**  yes  none | 2 (5.3%)  36 (94.7%) | 2 (7.7%)  24 (92.3%) |
| **Graft types**  *Quadriceps tendon*  *Hamstrings tendon*  *Patellar tendon*  *unknown* | 26 (68.4%)  8 (21.1%)  3 (7.9%)  1 (2.6%) | n.a.  n.a.  n.a.  n.a. |

Legend and abbreviations (Tab.A.1): ACL-R = group with ACL reconstruction; ACL-C = group with conservatively treated ACL rupture; n.a. = not applicable
