# Supplementary material for: Bilateral neuromuscular control in patients one year after unilateral ACL rupture or reconstruction. A cross-sectional study
Source: Heliyon. 2024 Jan 11;10(2):e24364. doi: 10.1016/j.heliyon.2024.e24364 (PMC10803901; doi:10.1016/j.heliyon.2024.e24364)
Supplement: Multimedia component 2 [file mmc2.docx]

Appendix Table A.2: Stair descent: Mean (standard deviations), p-values and effect sizes of normalized RMS values for the involved (injured) and non-involved (contralateral) limb of ACL-R and ACL-C participants, ACL-I with matched legs (based on side of injury) as controls in the 3 phases pre-activation (PRE), weight acceptance (WA) and push-off (PO).

| **Stair descent, pre-activity (PRE)** | | | | | | | | | | | | | | | | | |
| --- | --- | --- | --- | --- | --- | --- | --- | --- | --- | --- | --- | --- | --- | --- | --- | --- | --- |
| **Muscle** | **Group** | | | | | | **p-values** | | | | | | | | | | |
|  | **ACL-R** | | **ACL-C** | | **ACL-I = Control** | | **ACL-R** | **ACL-C** | **ACL-I** | **all 3 groups** | | **ACL-R vs. ACL-I** | | **ACL-C vs. ACL-I** | | **ACL-R vs. ACL-C** | |
|  | involved | non-involved | involved | non-involved | involved | non-involved | between legs | between legs | between legs | involved | non-involved | involved | non-involved | involved | non-involved | involved | non-involved |
|  | [1] | [2] | [3] | [4] | [5] | [6] | [1]vs[2]^ | [3]vs[4]^ | [5]vs[6]^ | overall~ | overall~ | [1]vs[5]° | [2]vs[6]° | [3]vs[5]° | [4]vs[6]° | [1]vs[3]° | [2]vs[4]° |
| **VM** | 128.5 (35.0) | 158.0 (63.1) | 116.9 (35.2) | 120.5 (47.4) | 114.1 (40.0) | 122.4 (49.0) | 0.004* | 0.619 | 0.566 | 0.328 | 0.020* | 0.209 | 0.011* | 0.875 | 0.978 | 0.199 | 0.030* |
|  |  |  |  |  |  |  | ES 0.34 |  |  |  |  |  | ES 0.30 |  |  |  | ES 0.27 |
| **VL** | 125.4 (27.2) | 149.0 (36.6) | 123.9 (24.2) | 133.1 (33.6) | 121.7 (44.0) | 107.8 (37.9) | 0.001* | 0.170 | 0.404 | 0.806 | < 0.0001* | 0.520 | < 0.0001* | 0.736 | 0.116* | 0.780 | 0.106 |
|  |  |  |  |  |  |  | ES 0.37 |  |  |  |  |  | ES 0.47 |  | ES 0.31 |  |  |
| **BF** | 99.0 (36.4) | 95.6 (38.0) | 105.9 (43.4) | 88.1 (42.0) | 113.4 (43.9) | 130.5 (49.2) | 0.765 | 0.174 | 0.014* | 0.362 | 0.001* | 0.170 | 0.002* | 0.652 | 0.001* | 0.369 | 0.403 |
|  |  |  |  |  |  |  |  |  | ES 0.29 |  |  |  | ES 0.36 |  | ES 0.42 |  |  |
| **ST** | 99.4 (36.9) | 95.4 (45.7) | 107.7 (36.0) | 99.4 (43.5) | 119.5 (46.2) | 125.0 (47.2) | 0.164 | 0.493 | 0.235 | 0.174 | 0.016* | 0.075 | 0.007* | 0.245 | 0.052 | 0.491 | 0.417 |
|  |  |  |  |  |  |  |  |  |  |  |  |  | ES 0.32 |  |  |  |  |
| **Stair descent, weight acceptance (WA)** | | | | | | | | | | | | | | | | | |
| **Muscle** | **Group** | | | | | | **p-values** | | | | | | | | | | |
|  | **ACL-R** | | **ACL-C** | | **ACL-I = Control** | | **ACL-R** | **ACL-C** | **ACL-I** | **all 3 groups** | | **ACL-R vs. ACL-I** | | **ACL-C vs. ACL-I** | | **ACL-R vs. ACL-C** | |
|  | involved | non-involved | involved | non-involved | involved | non-involved | between legs | between legs | between legs | involved | non-involved | involved | non-involved | involved | non-involved | involved | non-involved |
|  | [1] | [2] | [3] | [4] | [5] | [6] | [1]vs[2]^ | [3]vs[4]^ | [5]vs[6]^ | overall~ | overall~ | [1]vs[5]° | [2]vs[6]° | [3]vs[5]° | [4]vs[6]° | [1]vs[3]° | [2]vs[4]° |
| **VM** | 262.2 (92.4) | 339.2 (174.4) | 231.3 (67.5) | 257.4 (87.7) | 264.1 (118.8) | 242.4 (92.2) | 0.004* | 0.935 | 0.706 | 0.654 | 0.025* | 0.858 | 0.008* | 0.443 | 0.538 | 0.390 | 0.079 |
|  |  |  |  |  |  |  | ES 0.34 |  |  |  |  |  | ES 0.31 |  |  |  |  |
| **VL** | 247.8 (68.8) | 279.2 (82.9) | 247.0 (49.9) | 253.2 (71.2) | 228.3 (85.1) | 214.2 (76.3) | 0.003* | 0.568 | 0.474 | 0.331 | 0.016* | 0.228 | 0.007* | 0.179 | 0.083 | 0.988 | 0.243 |
|  |  |  |  |  |  |  | ES 0.35 |  |  |  |  |  | ES 0.31 |  |  |  |  |
| **BF** | 75.5 (28.4) | 75.8 (31.4) | 57.6 (21.0) | 85.0 (43.7) | 86.4 (55.9) | 86.9 (44.6) | 0.626 | 0.015* | 0.870 | 0.051 | 0.612 | 0.901 | 0.321 | 0.089 | 0.920 | 0.008* | 0.530 |
|  |  |  |  |  |  |  |  | ES 0.34 |  |  |  |  |  |  |  |  |  |
| **ST** | 46.2 (19.6) | 43.6 (16.7) | 46.2 (21.4) | 50.5 (25.2) | 57.4 (37.1) | 67.5 (51.8) | 0.612 | 0.903 | 0.993 | 0.693 | 0.308 | 0.442 | 0.171 | 0.495 | 0.507 | 0.970 | 0.281 |
|  |  |  |  |  |  |  |  |  |  |  |  |  |  |  |  |  |  |
| **Stair descent, push-off (PO)** | | | | | | | | | | | | | | | | | |
| **Muscle** | **Group** | | | | | | **p-values** | | | | | | | | | | |
|  | **ACL-R** | | **ACL-C** | | **ACL-I = Control** | | **ACL-R** | **ACL-C** | **ACL-I** | **all 3 groups** | | **ACL-R vs. ACL-I** | | **ACL-C vs. ACL-I** | | **ACL-R vs. ACL-C** | |
|  | involved | non-involved | involved | non-involved | involved | non-involved | between legs | between legs | between legs | involved | non-involved | involved | non-involved | involved | non-involved | involved | non-involved |
|  | [1] | [2] | [3] | [4] | [5] | [6] | [1]vs[2]^ | [3]vs[4]^ | [5]vs[6]^ | overall~ | overall~ | [1]vs[5]° | [2]vs[6]° | [3]vs[5]° | [4]vs[6]° | [1]vs[3]° | [2]vs[4]° |
| **VM** | 289.4 (136.0) | 365.6 (192.7) | 327.6 (149.0) | 331.8 (104.0) | 236.6 (112.0) | 258.2 (140.2) | 0.008* | 0.798 | 0.719 | 0.081 | 0.034* | 0.166 | 0.019* | 0.032* | 0.040* | 0.291 | 0.944 |
|  |  |  |  |  |  |  | ES 0.31 |  |  |  |  |  | ES 0.27 |  | ES 0.26 |  |  |
| **VL** | 253.6 (106.9) | 275.5 (93.4) | 255.4 (81.7) | 292.2 (73.2) | 202.3 (97.4) | 201.0 (85.5) | 0.018* | 0.063 | 0.994 | 0.069 | < 0.0001* | 0.067 | 0.002* | 0.039* | < 0.0001* | 0.637 | 0.357 |
|  |  |  |  |  |  |  | ES 0.28 |  |  |  |  |  | ES 0.36 |  | ES 0.48 |  |  |
| **BF** | 48.6 (18.3) | 54.3 (22.0) | 49.7 (25.4) | 64.1 (33.0) | 78.9 (42.2) | 77.0 (41.0) | 0.145 | 0.097 | 0.647 | 0.001* | 0.048* | 0.001* | 0.014* | 0.004* | 0.204 | 0.693 | 0.348 |
|  |  |  |  |  |  |  |  |  |  |  |  | ES 0.39 | ES 0.29 | ES 0.37 |  |  |  |
| **ST** | 52.5 (23.1) | 54.8 (24.4) | 58.2 (27.5) | 58.5 (28.1) | 65.2 (28.9) | 67.0 (41.3) | 0.481 | 0.954 | 0.911 | 0.146 | 0.670 | 0.048* | 0.414 | 0.341 | 0.647 | 0.443 | 0.584 |

Legend and abbreviations (Tab.A.2): ~Kruskal-Wallis test; ^Wilcoxon signed-rank test; °Mann-Whitney-U test; *indicate statistically significant differences between groups or legs (p<0.05); dashes indicate not applicable; ACL = anterior cruciate ligament; BF = biceps femoris; involved = injured leg, respective matched leg of controls; PO = push-off; PRE = pre-activity; RMS = root mean square; SD = standard deviation; ST = semitendinosus; non-involved = non-injured leg, respective contralateral leg; VM = vastus medialis; VL = vastus lateralis; WA = weight-acceptance
