# Supplementary material for: Bilateral neuromuscular control in patients one year after unilateral ACL rupture or reconstruction. A cross-sectional study
Source: Heliyon. 2024 Jan 11;10(2):e24364. doi: 10.1016/j.heliyon.2024.e24364 (PMC10803901; doi:10.1016/j.heliyon.2024.e24364)
Supplement: Multimedia component 3 [file mmc3.docx]

Appendix Table A.3: Reflex activity: Mean (standard deviations), p-values and effect sizes of normalized RMS values for the involved (injured) and non-involved (contralateral) limb of ACL-R and ACL-C participants, ACL-I with matched legs (based on side of injury) as controls in the 4 reflex windows pre-activation (PRE_50), short latency response (SLR), medium latency response (MLR) and long latency response (LLR).

| **Reflex activity, pre-activation 50ms (PRE_50)** | | | | | | | | | | | | | | | | | |
| --- | --- | --- | --- | --- | --- | --- | --- | --- | --- | --- | --- | --- | --- | --- | --- | --- | --- |
| **Muscle** | **Group** | | | | | | **p-values** | | | | | | | | | | |
|  | **ACL-R** | | **ACL-C** | | **ACL-I = Control** | | **ACL-R** | **ACL-C** | **ACL-I** | **all 3 groups** | **all 3 groups** | **ACL-R vs. ACL-I** | | **ACL-C vs. ACL-I** | | **ACL-R vs. ACL-C** | |
|  | involved | non-involved | involved | non-involved | involved | non-involved | between legs | between legs | between legs | involved | non-involved | involved | non-involved | involved | non-involved | involved | non-involved |
|  | [1] | [2] | [3] | [4] | [5] | [6] | [1]vs[2]^ | [3]vs[4]^ | [5]vs[6]^ | overall~ | overall~ | [1]vs[5]° | [2]vs[6]° | [3]vs[5]° | [4]vs[6]° | [1]vs[3]° | [2]vs[4]° |
| **VM** | 175.4 (194.9) | 168.5 (105.5) | 101.1 (37.0) | 110.8 (39.9) | 139.5 (67.1) | 133.2 (70.0) | 0.248 | 0.238 | 0.323 | 0.013* | 0.103 | 0.645 | 0.207 | 0.013* | 0.312 | 0.007* | 0.038* |
|  |  |  |  |  |  |  |  |  |  |  |  |  |  | ES 0.31 |  | ES 0.35 |  |
| **VL** | 155.8 (103.7) | 171.9 (103.6) | 122.8 (35.1) | 130.2 (50.9) | 146.7 (56.7) | 134.6 (44.1) | 0.105 | 0.603 | 0.307 | 0.229 | 0.338 | 0.723 | 0.344 | 0.081 | 0.577 | 0.232 | 0.150 |
|  |  |  |  |  |  |  |  |  |  |  |  |  |  |  |  |  |  |
| **BF** | 47.6 (37.9) | 56.7 (40.7) | 42.2 (27.6) | 69.6 (55.8) | 66.2 (41.3) | 73.5 (48.3) | 0.614 | 0.061 | 0.405 | 0.035* | 0.336 | 0.035* | 0.136 | 0.022* | 0.596 | 0.854 | 0.450 |
|  |  |  |  |  |  |  |  |  |  |  |  | ES 0.25 |  | ES 0.29 |  |  |  |
| **ST** | 60.4 (29.5) | 60.2 (27.1) | 59.6 (28.8) | 58.1 (28.9) | 46.3 (31.4) | 53.3 (38.7) | 0.726 | 0.713 | 0.084 | 0.099 | 0.413 | 0.040* | 0.176 | 0.121 | 0.436 | 0.964 | 0.819 |
|  |  |  |  |  |  |  |  |  |  |  |  |  |  |  |  |  |  |
| **Reflex activity, short latency response (SLR)** | | | | | | | | | | | | | | | | | |
| **Muscle** | **Group** | | | | | | **p-values** | | | | | | | | | | |
|  | **ACL-R** | | **ACL-C** | | **ACL-I = Control** | | **ACL-R** | **ACL-C** | **ACL-I** | **all 3 groups** |  | **ACL-R vs. ACL-I** | | **ACL-C vs. ACL-I** | | **ACL-R vs. ACL-C** | |
|  | involved | non-involved | involved | non-involved | involved | non-involved | between legs | between legs | between legs | involved | non-involved | involved | non-involved | involved | non-involved | involved | non-involved |
|  | [1] | [2] | [3] | [4] | [5] | [6] | [1]vs[2]^ | [3]vs[4]^ | [5]vs[6]^ | overall~ | overall~ | [1]vs[5]° | [2]vs[6]° | [3]vs[5]° | [4]vs[6]° | [1]vs[3]° | [2]vs[4]° |
| **VM** | 262.0 (223.9) | 300.5 (274.2) | 194.6 (132.7) | 192.5 (113.2) | 191.4 (104.2) | 137.1 (74.5) | 0.728 | 0.990 | 0.004* | 0.582 | 0.011* | 0.427 | 0.004* | 0.823 | 0.073 | 0.332 | 0.260 |
|  |  |  |  |  |  |  |  |  | ES 0.34 |  |  |  | ES 0.34 |  |  |  |  |
| **VL** | 209.7 (136.7) | 223.6 (155.1) | 171.4 (90.0) | 179.0 (95.8) | 166.5 (74.0) | 160.0 (94.0) | 0.544 | 0.568 | 0.359 | 0.643 | 0.124 | 0.401 | 0.039* | 0.988 | 0.356 | 0.449 | 0.387 |
|  |  |  |  |  |  |  |  |  |  |  |  |  |  |  |  |  |  |
| **BF** | 260.0 (373.4) | 242.8 (233.8) | 198.6 (142.2) | 210.6 (156.9) | 206.5 (145.9) | 192.4 (162.1) | 0.922 | 0.946 | 0.272 | 0.570 | 0.769 | 0.307 | 0.551 | 0.557 | 0.521 | 0.645 | 0.977 |
|  |  |  |  |  |  |  |  |  |  |  |  |  |  |  |  |  |  |
| **ST** | 239.5 (222.9) | 183.4 (144.4) | 206.0 (164.5) | 181.8 (160.5) | 233.1 (305.0) | 174.2 (201.5) | 0.166 | 0.278 | 0.149 | 0.351 | 0.490 | 0.169 | 0.205 | 0.312 | 0.693 | 0.818 | 0.626 |
|  |  |  |  |  |  |  |  |  |  |  |  |  |  |  |  |  |  |
| **Reflex activity, medium latency response (MLR)** | | | | | | | | | | | | | | | | | |
| **Muscle** | **Group** | | | | | | **p-values** | | | | | | | | | | |
|  | **ACL-R** | | **ACL-C** | | **ACL-I = Control** | | **ACL-R** | **ACL-C** | **ACL-I** | **all 3 groups** |  | **ACL-R vs. ACL-I** | | **ACL-C vs. ACL-I** | | **ACL-R vs. ACL-C** | |
|  | involved | non-involved | involved | non-involved | involved | non-involved | between legs | between legs | between legs | involved | non-involved | involved | non-involved | involved | non-involved | involved | non-involved |
|  | [1] | [2] | [3] | [4] | [5] | [6] | [1]vs[2]^ | [3]vs[4]^ | [5]vs[6]^ | overall~ | overall~ | [1]vs[5]° | [2]vs[6]° | [3]vs[5]° | [4]vs[6]° | [1]vs[3]° | [2]vs[4]° |
| **VM** | 431.8 (409.8) | 380.3 (257.8) | 265.8 (147.3) | 249.3 (128.3) | 329.5 (177.8) | 276.6 (188.9) | 0.376 | 0.797 | 0.033* | 0.097 | 0.078 | 0.645 | 0.092 | 0.137 | 0.888 | 0.025* | 0.027* |
|  |  |  |  |  |  |  |  |  | ES 0.25 |  |  |  |  |  |  |  |  |
| **VL** | 385.1 (247.6) | 365.4 (218.5) | 300.5 (125.9) | 341.5 (212.8) | 313.3 (165.4) | 270.9 (186.3) | 0.765 | 0.264 | 0.008* | 0.472 | 0.069 | 0.317 | 0.030* | 0.814 | 0.119 | 0.284 | 0.503 |
|  |  |  |  |  |  |  |  |  | ES 0.31 |  |  |  |  |  |  |  |  |
| **BF** | 258.2 (162.4) | 320.4 (257.0) | 262.4 (137.7) | 329.8 (204.0) | 377.2 (312.4) | 317.9 (299.5) | 0.400 | 0.238 | 0.044* | 0.325 | 0.504 | 0.167 | 0.494 | 0.276 | 0.258 | 0.737 | 0.568 |
|  |  |  |  |  |  |  |  |  | ES 0.23 |  |  |  |  |  |  |  |  |
| **ST** | 197.6 (181.0) | 196.0 (144.6) | 213.5 (122.1) | 269.3 (271.3) | 461.5 (454.3) | 409.5 (440.9) | 0.808 | 0.510 | 0.145 | 0.011* | 0.146 | 0.007* | 0.050* | 0.037* | 0.272 | 0.224 | 0.577 |
|  |  |  |  |  |  |  |  |  |  |  |  | ES 0.33 |  | ES 0.27 |  |  |  |
| **Reflex activity, long latency response (LLR)** | | | | | | | | | | | | | | | | | |
| **Muscle** | **Group** | | | | | | **p-values** | | | | | | | | | | |
|  | **ACL-R** | | **ACL-C** | | **ACL-I = Control** | | **ACL-R** | **ACL-C** | **ACL-I** | **all 3 groups** |  | **ACL-R vs. ACL-I** | | **ACL-C vs. ACL-I** | | **ACL-R vs. ACL-C** | |
|  | involved | non-involved | involved | non-involved | involved | non-involved | between legs | between legs | between legs | involved | non-involved | involved | non-involved | involved | non-involved | involved | non-involved |
|  | [1] | [2] | [3] | [4] | [5] | [6] | [1]vs[2]^ | [3]vs[4]^ | [5]vs[6]^ | overall~ | overall~ | [1]vs[5]° | [2]vs[6]° | [3]vs[5]° | [4]vs[6]° | [1]vs[3]° | [2]vs[4]° |
| **VM** | 407.1 (480.0) | 311.2 (215.8) | 220.8 (151.1) | 215.1 (140.0) | 213.3 (141.6) | 196.4 (152.8) | 0.741 | 0.412 | 0.208 | 0.034* | 0.023* | 0.021* | 0.008* | 0.824 | 0.444 | 0.034* | 0.082 |
|  |  |  |  |  |  |  |  |  |  |  |  | ES 0.28 | ES 0.31 |  |  | ES 0.28 |  |
| **VL** | 287.4 (187.2) | 307.2 (209.9) | 235.8 (160.8) | 219.0 (147.1) | 205.1 (119.5) | 167.8 (89.9) | 0.952 | 0.439 | 0.066 | 0.110 | 0.005* | 0.040* | 0.001* | 0.645 | 0.289 | 0.165 | 0.074 |
|  |  |  |  |  |  |  |  |  |  |  |  |  | ES 0.38 |  |  |  |  |
| **BF** | 204.5 (128.6) | 247.9 (220.6) | 228.3 (117.6) | 265.1 (127.2) | 228.0 (124.7) | 243.9 (171.5) | 0.586 | 0.104 | 0.900 | 0.624 | 0.368 | 0.417 | 0.983 | 0.883 | 0.189 | 0.399 | 0.231 |
|  |  |  |  |  |  |  |  |  |  |  |  |  |  |  |  |  |  |
| **ST** | 254.6 (222.0) | 191.5 (172.0) | 306.3 (211.3) | 228.0 (173.4) | 231.3 (159.4) | 247.9 (233.1) | 0.091 | 0.122 | 0.900 | 0.457 | 0.340 | 0.963 | 0.166 | 0.209 | 0.911 | 0.347 | 0.284 |
|  |  |  |  |  |  |  |  |  |  |  |  |  |  |  |  |  |  |

Legend and abbreviations (Tab.A.3): ~Kruskal-Wallis test; ^Wilcoxon signed-rank test; °Mann-Whitney-U test; *indicate statistically significant differences between groups or legs (p<0.05); dashes indicate not applicable; ACL = anterior cruciate ligament; BF = biceps femoris; involved = injured leg, respective matched leg of controls; PO = push-off; PRE_50 = pre-activation 50ms before; RMS = root mean square; SD = standard deviation; ST = semitendinosus; non-involved = non-injured leg, respective contralateral leg; VM = vastus medialis; VL = vastus lateralis
